# Supplementary figures and images for: Radial alignment of microtubules through tubulin polymerization in an evaporating droplet
Source: PLoS One. 2020 Apr 10;15(4):e0231352. doi: 10.1371/journal.pone.0231352 (PMC7147791; doi:10.1371/journal.pone.0231352)

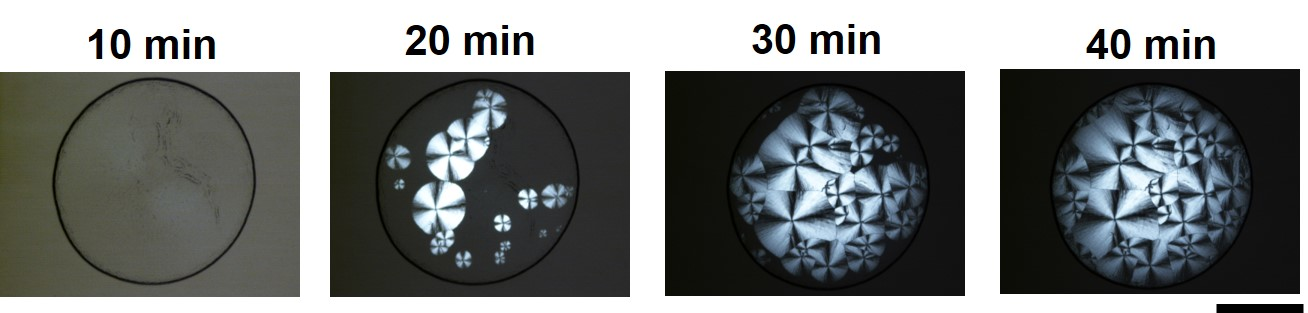

Supplement: S1 Fig — Time-lapse polarized optical microscopy images of MT spherulites by evaporating tubulin solution at 37 oC in a thermostatic chamber. The volume of tubulin solution was used 2 μL for placing the droplet and concentration was 180 μM. Scale bar: 1 mm. (TIF) [file pone.0231352.s001.tif]

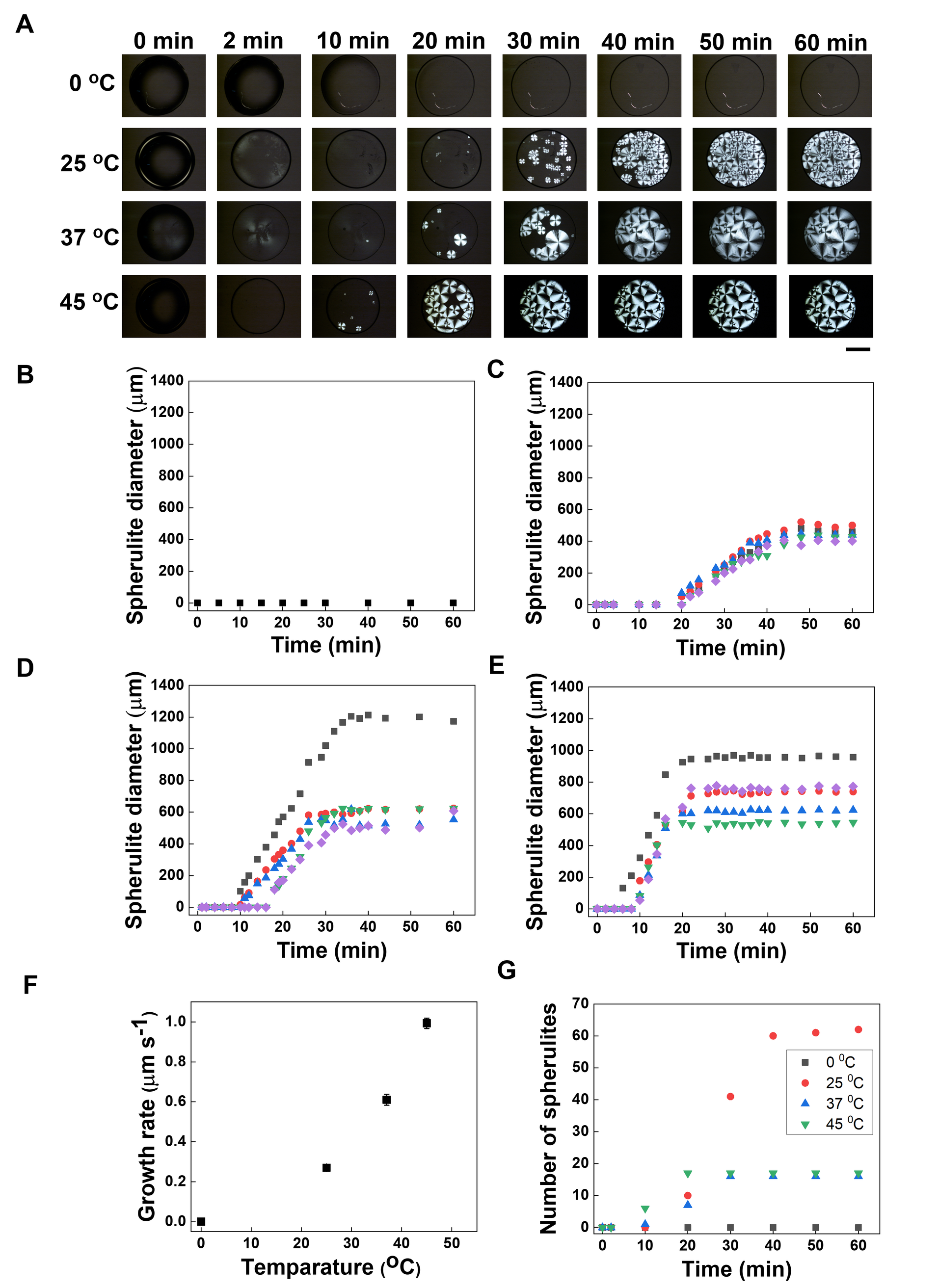

Supplement: S2 Fig — (A) Time lapse polarized optical microscopy images of droplets of tubulin solution at different temperatures; 0 °C, 25 °C, 37 °C, 45 °C. Scale bar: 1 mm. The volume of tubulin solution was used 2 μL for placing the droplets and concentration was 180 μM. Growth of spherulite diameter with time at (B) 0 °C, (C) 25 °C, (D) 37 °C and (E) 45 °C. The different color indicates different spherulites and number of spherulites was considered five in each case. (F) Growth rate of spherulites at different temperatures. Error bar: s.d. The growth rate was determined from the slopes of the steeper region of the plots of diameter versus time. From the average of growth rate of five spherulites, the growth rate was estimated for each temperature. (G) And number of spherulites with time at different temperatures. (TIF) [file pone.0231352.s002.tif]

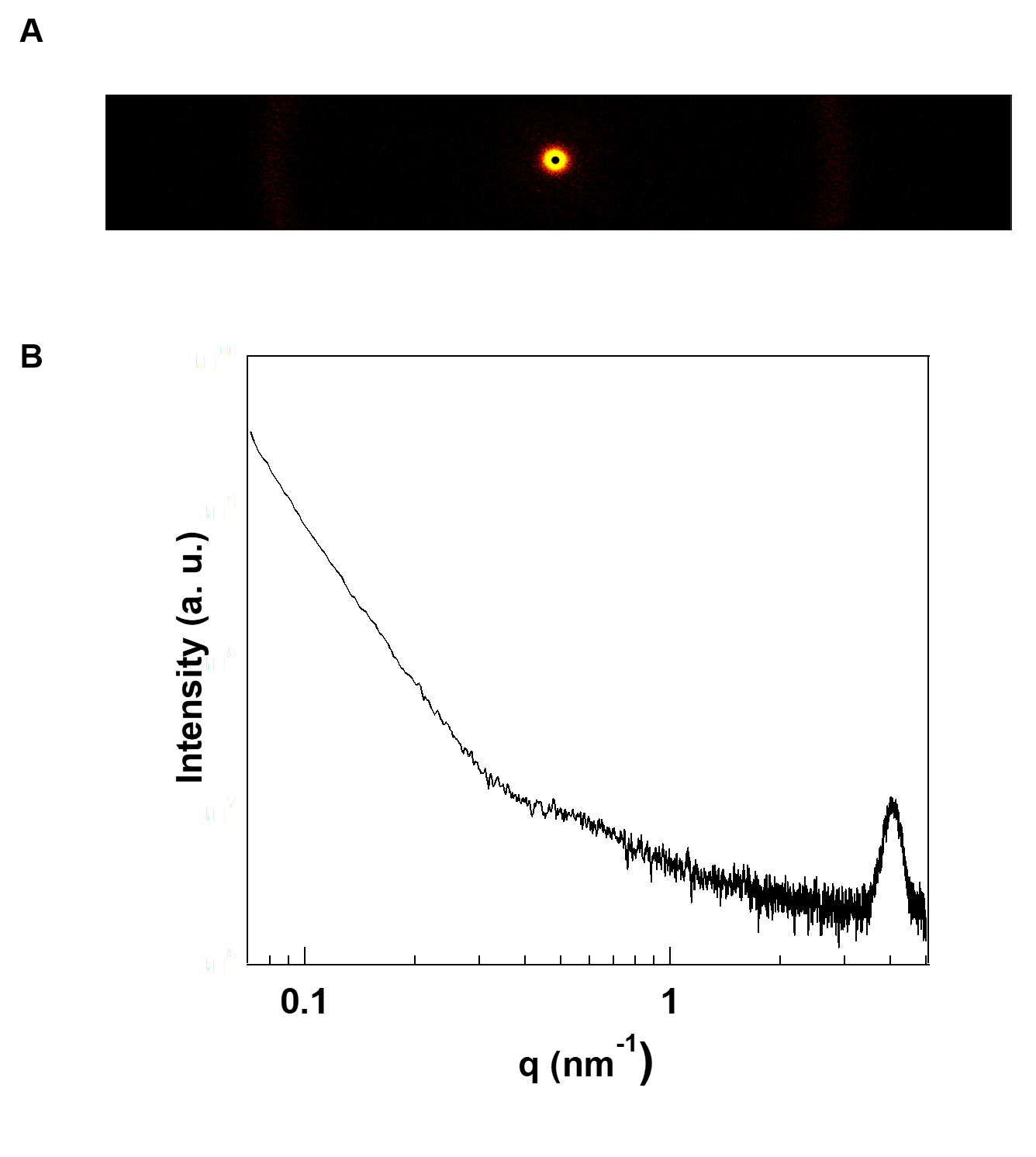

Supplement: S3 Fig — (A) Two-dimensional scattering from the droplet of buffer solution and (B) profile of scattering curve. The peak corresponds to the background coming from the sample holder. The volume of buffer solution (80 mM PIPES, 1 mM EGTA, 1 mM MgCl2) was used 2 μL for placing the droplet. (TIF) [file pone.0231352.s003.tif]
